# Muscle Strength and Joint Range of Motion of the Spine and Lower Extremities in Female Prepubertal Elite Rhythmic and Artistic Gymnasts

**DOI:** 10.3390/jfmk8040153

**Published:** 2023-11-02

**Authors:** Athanasios Mandroukas, Ioannis Metaxas, Yiannis Michailidis, Thomas Metaxas

**Affiliations:** Laboratory of Evaluation of Human Biological Performance, Department of Physical Education and Sport Sciences, Aristotle University of Thessaloniki, 57001 Thessaloniki, Greece; amandrou@phed.auth.gr (A.M.); metaxasi@phed-sr.auth.gr (I.M.); ioannimd@phed.auth.gr (Y.M.)

**Keywords:** rhythmic gymnastics, artistic gymnastics, muscle strength, range of motion, back, lower extremities

## Abstract

The purpose of this study was to investigate and compare the passive joint range of motion (PROM) and muscle strength in prepubertal rhythmic gymnasts (RGs), artistic gymnasts (AGs), and a control group (CG) of the same age. A total of 54 prepubertal girls were divided into three groups: 18 RGs (age 11.14 ± 0.7, height 142.6 ± 5.81, and body mass 31.2 ± 3.63); 18 AGs (age 11.27 ± 0.99, height 139.6 ± 5.85, and body mass 31.7 ± 3.21), and 18 school girls who are defined as CG (age 10.55 ± 0.42, height 145.33 ± 6.95, and body mass 42.1 ± 8.21) participated in the study. All athletes were elites and participated in national competitions. The CG participated only in their school physical education program. Isokinetic peak torques were measured using an isokinetic dynamometer (Cybex II) at 60, 180, and 300°·sec^−1^. Body mass index was greater in the CG compared to RGs and AGs (*p* < 0.001). PROM in cervical extension in RG was significantly higher compared to the AG and CG (*p* < 0.001). The athlete groups, RG and AG, showed significantly greater PROM in knee flexion (*p* < 0.001), hip flexion (*p* < 0.001), and hip abduction (*p* < 0.05) compared to CG. PROM in hip flexion was different between the left and right leg in RGs. The relative muscle strength of the quadriceps in the RG and AG was significantly greater compared to CG (*p* < 0.001 and *p* < 0.01 respectively). Gymnastics training in prepubertal ages can improve neuromuscular function and increase the relative muscle strength. Therefore, it is essential to note that when evaluating children within the developmental ages, especially those involved in sports, the type of muscle strength to be assessed should be specified.

## 1. Introduction

Children’s physical activity is increasingly characterized by the specialization in a specific sport. Furthermore, the sport requirements and competition have been increased significantly as suggested by the high intensity of the workouts and the frequency of the competitions. With respect to rhythmic gymnastics and artistic gymnastics, the selection of the girls is made from a very young age. Age is an important factor for the selection of athletes in these sports, which require muscle strength, large range of motion, and the combination of these, in order to achieve maximal performance [1,2]. The long and intense training of female athletes can cause, among other things, qualitative changes in the neuromuscular function with favorable conditions for explosive power and vitality [3,4]. At this age the skeleton is soft, and the endurance of the tendons and ligaments is greater than the endurance of the skeleton [5]. As a consequence of improved neuromuscular adaptations, the functional training in rhythmic gymnasts (RGs) and artistic gymnasts (AGs) leads to the correct technique of their particular sports, because, even at these ages, there are changes in the muscle architecture (penal angle), increased coordination, and balance between prime mover or agonist and antagonist muscles [6,7]. The aforementioned are sine qua non to achieve great performances, but, at the same time, they expose the athletes to a great risk of possible repetitive strain injury [8,9].

Rhythmic gymnastics as an artistic sport is highly demanding in the complexity of skills. Exercise in this sport involves coordinating different body parts with the apparatus: the ball, hoop, club, ribbon, and rope. A gymnast might achieve excellent performance when she is able to execute the specific exercises indicating her physical abilities at the best level and showing mastery of the special apparatus movements required by the international Code of Points [10]. Artistic gymnastics is a complex sport consisting of technical skills in the events of floor exercise, the uneven bars, the balance beam, and the vault. Training in both rhythmic and artistic gymnastics requires repetitive and long-lasting workouts in the fundamental elements and the basic positions [11,12] demanding the coordination of handling various apparatus. Muscle strength and power, muscle endurance, as well as large range of motion are required to attain positions not seen very often in sports. Other studies have associated the risk of developing lower back complaints with the specific sports that demand repetitive or high velocity twisting or repetitive bending flexibility, particularly in extension [13,14]. Athletes participating in sports such as rhythmic and artistic gymnastics have been shown to be at increased risk of developing lower back complaints [14,15,16,17,18,19]. The kind of movements of RGs and AGs require high muscle strength of lower extremities, abdominals, and back muscles. Sufficient muscle strength of the hamstrings and quadriceps is a prerequisite for female athletes to learn proper movement technique and meet the demands of the sport. Conversely, insufficient (decreased) muscle strength can lead to incorrect technique which can negatively affect performance and increase the risk of injury [20]. Furthermore, when there is an imbalance in the relationship between joint range of motion and muscle power, the optimal movement pattern is disrupted and the posture of the spine will be affected, thereby increasing the risk of injury [20,21]. To the best of our knowledge there are few studies examining isokinetic torque and joint passive range of motion (PROM) including RGs compared to AGs and non-exercisers (untrained girls). Thus, the purpose of this study was to examine and compare the joint mobility and muscle strength of the spine and lower extremities in prepubertal elite athletes in rhythmic gymnastics, artistic gymnastics, and untrained participants.

## 2. Materials and Methods

### 2.1. Participants

The power analysis was conducted prior to the study being performed, based on previous studies of similar research design [22,23]. An effect size of >0.6, a probability error of 0.05, and a power of 0.95 were used for the 3 groups. Those indicated that 48 participants was the smallest acceptable number of participants to analyze the interaction. The calculations for effect size (ES) and statistical power were performed using G*Power software: Statistical Power Analyzes for Windows, Version 3.1.9.7 according to Cohen’s f criteria [24]. This study involved 54 girls, divided into 3 groups: 18 RGs, 18 AGs, and 18 school girls who are defined as the control group (CG) participated in the study. All athletes participated in national competitions. CG participated only in their school’s physical education program (2 to 3 40 min weekly classes), which consisted of mainly ball-games, stretching exercises, some calisthenics, and did not take part in any other sport activities in organized form. Basic anthropometric data of the groups are shown in Table 1.

Participants visited the laboratory on two occasions one day apart. The first visit was an orientation session that included anthropometric assessments, measurements of the joint PROM, and also a questionnaire that included their relevant physical and medical profile. In the second visit the participants were tested on the isokinetic dynamometer. After 5 min rest in the supine position, the heart rate (HR) using a monitor (Polar Electro, Sweden) and the blood pressure (BP) using a cuff on the left arm were recorded.

Participants reported no musculoskeletal injuries of the lower limbs that would prevent them from performing maximal isokinetic contractions. None of the participants had been doing progressive resistive exercise the previous day before the testing. Participants underwent a though-knee examination before the test. All participants and their parents were informed of the nature, purpose, procedures, potential discomfort, risks, and benefits involved in the study before giving their voluntary written consent for participation. All participants completed a questionnaire that included their relevant medical and physical history. No participant was taking any medication prior to the study that might affect the results of the experiment. This study has been approved by the Institutional Review Board of the Exercise Physiology and Sport Rehabilitation Laboratory, Thessaloniki, Greece (No. 02/2021) and was in accordance with the Declaration of Helsinki.

### 2.2. Testing Procedures—Measurements of the Joint PROM

Standing height was measured without shoes to the nearest 1.0 cm, using a stadiometer (model 220, Seca, Hamburg, Germany). Body mass was measured to the nearest 0.1 kg using an electronic digital scale (model 770, Seca), with the participants wearing only training shorts. The body mass index (BMI) was calculated as the ratio of body mass in kg to the square of the standing body height in m (kg/m^2^).

Joint PROM was tested by two experienced physical therapists that paid special attention in the way the movement began, its stability, as well as the direction of the movement. No warm-up exercises were performed prior to the testing procedures. Both legs were measured and the PROM was recorded. The measurements were taken in a quiet room and all groups were measured for standing height, body mass, and mobility of the spine, hip, and knee joints. Testing procedures were conducted between 11 a.m. and 2 p.m., and the environment temperature was around 22 °C. Participants performed a 5 min warm-up only before muscle strength measurement.

Hip abduction was measured using a Lafayette Gollehon extendable goniometer [25,26], and the rest of the movements were conducted using the Myrin goniometer (Lic Rehab, 17183 Solna, Sweden), which has shown advantages in clinical trials [27] and is reliable [28]. The position of the goniometer was standardized in relation to the anatomical landmarks. All measurements were made on an adjustable bench and were evaluated passively without causing pain at the same time of the day. Three repetitions of the test were carried out for each joint and the highest value was recorded. Joint mobility of the lower extremity was tested in the hip flexion, knee flexion, and hip abduction. In the spine the PROM of the cervical, thoracic, and lumbar spine were examined. Before measuring the articular mobility of the spine in each participant, all the spinous processes from the seventh cervical vertebra (C7) to the first sacral vertebrae (S1) were marked with a red pen. In addition, a straight line in the posterior superior iliac spines and the anterior superior iliac crests was also drawn.

#### 2.2.1. Knee Flexion (Test of the Quadriceps Femoris)

The participant was laid in the prone position on the examination bench. The ankle joint was in plantar flexion just outside the bench. The pelvis was immobilized with a Velcro band to be in constant with the examination bench. The Myrin goniometer was placed 5 cm above the lateral malleolus and adjusted to zero. The examiner used his hand to apply straight equal pressure to the participant’s ankle so as to avoid an inward turn of the hip (if the direction of the movement is wrong, the leg extremity that is the ankle joint and the foot would move to the other side of the gluteus maximus muscle). The examiner’s pressure was such as to lead the joint through its largest possible orbit without causing pain [29,30]. The other test leader read and took note of the passive joint flexibility, otherwise known as the personal PROM. There were three separate measurements for each participant with small breaks in between. The largest orbit was then recorded.

#### 2.2.2. Hip Flexion—Straight Leg Raising Test (SLR) (Test of the Hamstring Muscles)

The participant was laid in the supine position on the examination bench; the goniometer was strapped to the lateral side of the thigh 5 cm above the patella and was adjusted to zero. Velcro bands immobilized the pelvis and the opposite leg. The examiner moved one of the participant’s legs to his shoulder and asked the participant to relax. The examiner, with one hand, stabilized the ankle joint not allowing the internal rotation of the hip and placed the other hand on the participant’s straight knee [30,31]. The second examiner recorded the results of any passive movement. Both examiners measured the final orbit of motion.

#### 2.2.3. Hip Abduction (Test of the Adductor Muscles)

With the participant supine on a bench, the Lafayette Gollehon extendable goniometer was placed on a transverse line connecting the anterior superior iliac spines. The fulcrum was adjusted over the iliac spine and the movable arms were strapped to the thighs above the patella. The pelvis was stabilized with a special belt when measuring to keep it still. The hips were in full abduction and knees straight [30].

### 2.3. Testing Procedures—Mobility of the Spine

#### 2.3.1. Trunk flexion (Stibor Test)

**Figure 1:**
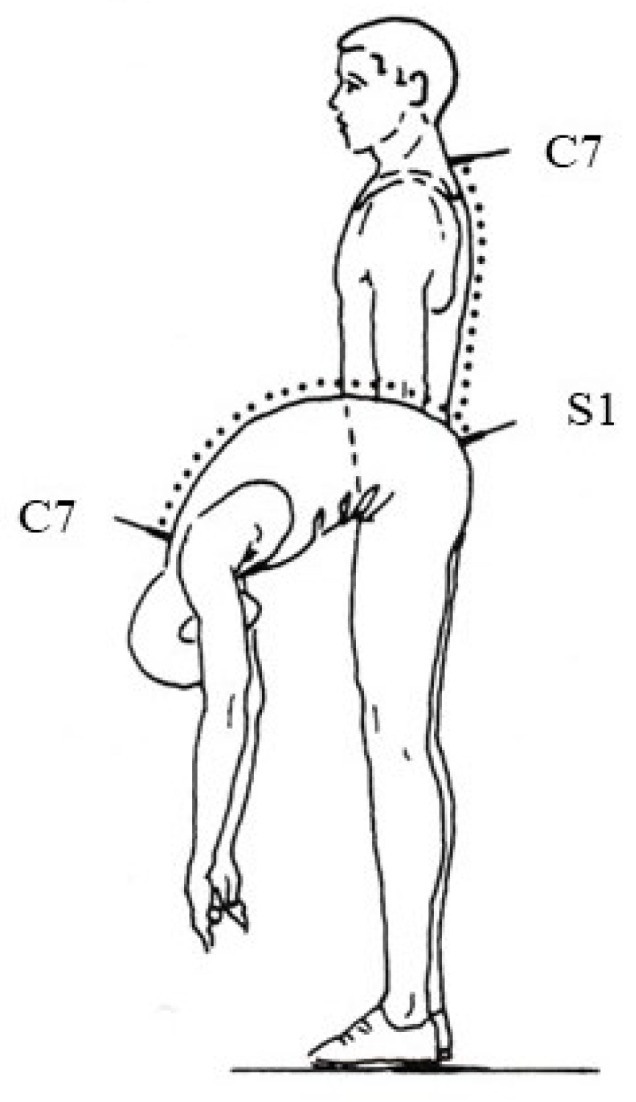
Measurement of the trunk flexion from the upright position (Stibor test). The identification of the seventh cervical vertebra (C7) to the first sacral vertebra (S1).

The participant was in the upright position. The identification of the spinous process of the C7 was made after palpation of the 6th and 7th cervical vertebrae. When the head was extended the spinous process of the 6th cervical vertebra could no longer be palpated, whereas the C7 could be palpated. The one end of the metering was placed in the spinous process of C7 and the other on the spinous process of S1. The participant, with stretched knees, hands free, and head relaxed, bends the trunk forward [30,32,33]. In full flexion of the trunk the increased difference was measured (Figure 1).

#### 2.3.2. Cervical Spine

The measurements were performed in 3 planes of movement: flexion—extension, lateral flexion, and axial rotation to both right and left side.

#### 2.3.3. Flexion and Extension of the Cervical Spine

The participant was in a seated position with trunk and head in a straight position. A solid ribbon with Myrin goniometer attached was placed around the head at the point just above the ear. The index of the goniometer pointed to zero. The examiner, giving instructions for the proper movement of the head, stabilized the trunk with once hand while the other hand lightly pressed the participant’s head forward. The extension of the head was made from the same starting point, with the trunk stabilized in a similar manner and the goniometer mounted at the same point [27,34].

#### 2.3.4. Lateral Flexion of the Cervical Spine

The participant sat with a straight trunk and head. The stabilizing ribbon was tied around the head, while the Myrin goniometer was placed on the forehead. The index of the goniometer pointed to zero. The examiner stabilized the shoulder with one hand while the other hand lightly pressed the participant’s head in the extreme left position (left lateral bending) and then right (right lateral bending). During the lateral bending the participant was instructed to avoid turning the head [27,34].

#### 2.3.5. Rotation of the Cervical Spine

The participant was either seated with a straight trunk and head in the supine position or lying down. The measurement was performed from both starting positions and no differences were found. The adhesive ribbon was placed around the head and the goniometer at the top of it. The index of the goniometer pointed to zero. The examiner stabilized the trunk of the participant and pushed the head slightly to the extreme rotating position [27,34]. The assistant recorded the cervical rotating (left and right) mobility.

#### 2.3.6. Lateral Flexion of the Trunk

**Figure 2:**
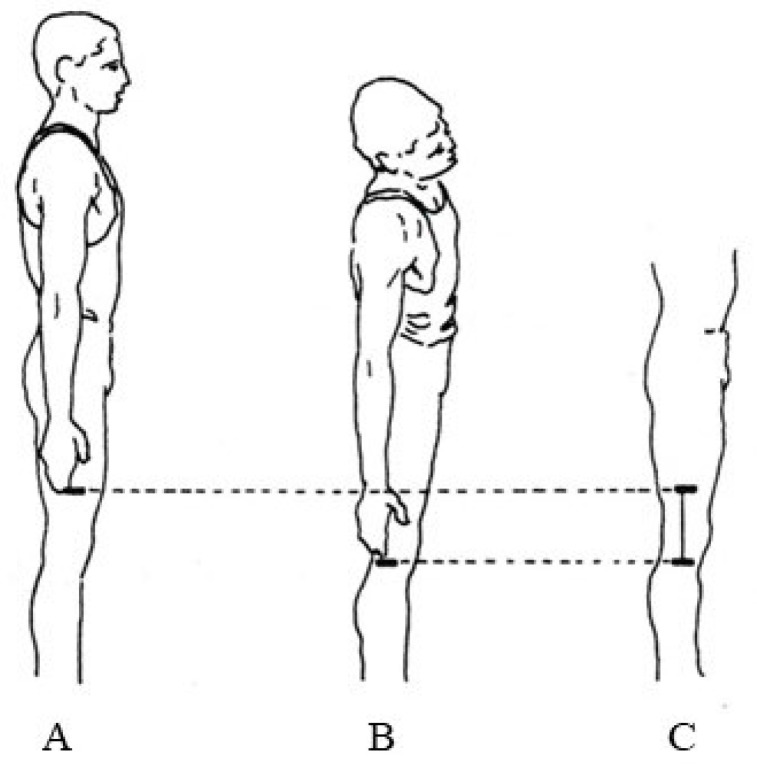
Lateral flexion of the trunk from the upright position. The distance difference between the two points (**A**) and (**B**) was measured (**C**).

The participant was in the upright position with the legs slightly stretched. From this position with the arms stretched and attached to the body, the contact point of the middle finger on the thigh (left and right) was recorded with a pen. After left and right trunk flexion, the contact point of the middle finger on the thigh was re-registered. The distance difference between the two points was measured. During bending the examiner controlled the movement of the participant so as to avoid rotating the torso and pelvis [35,36]. This measurement was performed for the mobility of the thoraco–lumbar junction (Figure 2).

#### 2.3.7. Lumbar Spine (Shober Test)

**Figure 3:**
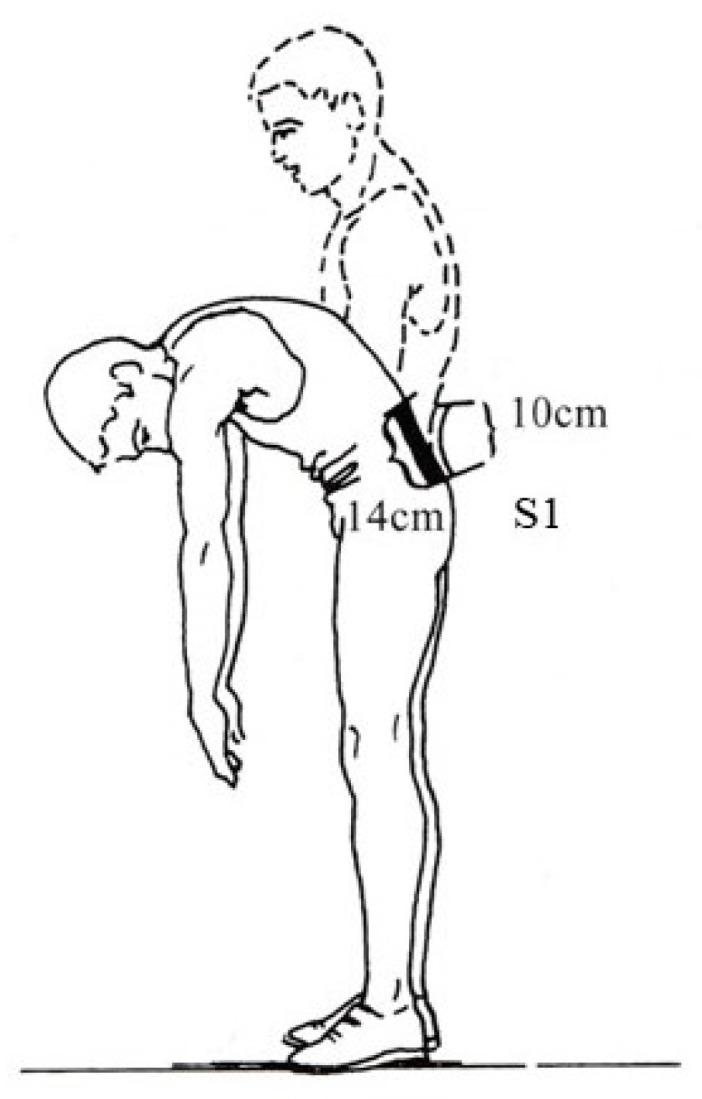
Measurement of the flexion of the lumbar spine (Schober test). From the standing position the distance from the S1 to 10 cm upwards was measured. In trunk flexion in this distance was increased by 4–5 cm.

The participant was in the standing position. The examiner, who was standing behind the participant, identified the posterior superior iliac spines using palpation and marked a straight line corresponding to the height of the spinous process of the 4th sacral vertebra. To secure the neutral position of the pelvis, the upper anterior iliac spines in the anterior part of the body were also marked by the examiner. Then, from the spinous process of the S1 was measured 10 cm upwards, i.e., to the level of the 1st and 2nd lumbar vertebrae (L1–L2). In trunk flexion with knees stretched, the difference will be increased by about 4–5 cm (Figure 3) [30,32,33,37].

#### 2.3.8. Thoracic Spine (Ott Test)

**Figure 4:**
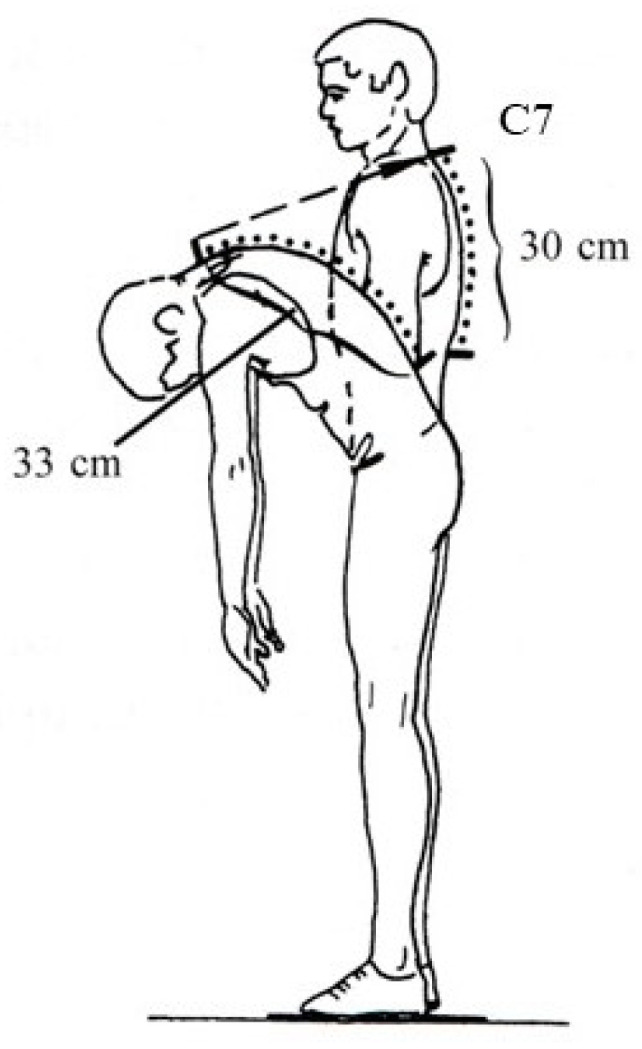
Measurement of the flexion of the thoracic spine (Ott test). The mobility of the thoracic spine was measured from C7 and 30 cm downwards in standing and bending positions. The difference between positions was increased by about 3 cm.

The participant was in the standing position. The mobility of the thoracic spine was measured from the spinous process of the C7 and 30 cm downwards. Then, the same distance was measured with the participant in a bending position, where the difference in flexion between positions will be increased by about 3 cm [30,32,38] (Figure 4).

### 2.4. Concentric Isokinetic Muscle Strength Measurements

Peak torque was measured using a speed controlled isokinetic dynamometer (Cybex II, Lumex Inc., Ronkonkoma, NY, USA), with a specially designed program which included torque comparison adjusted to the weight of the leg. Prior to the testing session, participants followed a standardized warm-up on a cycle ergometer (Monark 839, Varberg, Sweden) for 5 min with low resistance at 60 rev/min, prior to all strength measurements. This exercise was followed by a 5 min partial passive stretching of the knee flexors and extensors according to Mandroukas et al. [22] and the unilateral concentric muscle strength of the dominant leg was measured. The leg used most frequently for kicking the ball was identified as the dominant leg. The factors for the evaluation of strength performance were the absolute peak torque (APT) and the relative peak torque (RPT). APT is defined as the best value from all repetitions and RPT in relative to body mass values, for every type of movement and velocity.

#### Testing Protocol

For each angular velocity peak isokinetic torque was recorded simultaneously and the torque generated by the limb weight and the dynamometer arm was extracted from the obtained data. The participants were sitting on the chair of the dynamometer with stabilization straps at the trunk, thigh, and tibia to prevent extraneous joint movement. The knee to be tested was positioned at 90° of flexion (0° corresponding to fully extended knee) to align the axis of the dynamometer lever arm with the distal point of the lateral femoral condyle. The length of the lever arm was individually determined, and the resistance pad was placed at 5 cm above the malleoli. The non-tested leg was hanging freely. Knee extension started when the knee was positioned at 90° of flexion, while the knee flexion started when the knee was in full extension (0°). Alignment with an electronic goniometer (Lafayette Instrument Company, Indiana) was used for accuracy of the knee angle positioning and alignment of the joint prior to and during testing sessions. All participants, prior to the commencement of the testing, were familiarized with the isokinetic movements by performing several submaximal contractions under the guidance of the investigators. Participants were instructed to kick the leg as hard and as fast as they could through a complete ROM. Verbal encouragement was given during every trial. Participants were instructed to hold their arms comfortably across their chest to further isolate knee flexion and extension movements. Three repetitions were carried out at each angular velocity, and the best torque value was used. The trial proceeded from the high angular velocity to the low angular velocity. A 30 s rest period between each velocity was given and 60 sec rest period between each velocity measurement. Maximal isokinetic strength was recorded as the torque of the quadriceps and hamstring muscles throughout the whole ROM, at angular velocities of 60, 180, and 300°·s^−1^. The concentric strength ratio between the knee flexors and the knee extensors (H:Q ratio) was expressed as the ratio between the peak values at each velocity. The conventional H:Q ratio was calculated by dividing each participant’s highest concentric PT leg flexion by the highest concentric PT leg extension.

### 2.5. Statistical Analysis

Statistical analysis was undertaken using SPSS V.26.0 (SPSS Inc., Chicago, IL, USA). Initially, descriptive statistics were used to calculate means and standard deviations for the testing sessions, for all groups. One-way analysis of variance (ANOVA) and post hoc analysis (Scheffé test) were used to determine which groups in the ANOVA differed from each other. Effect sizes for variance analyses were given as partial eta squared (η_p_^2^) with values ≥0.01, ≥0.06, and ≥0.14 indicating small, moderate, or large effects, respectively [24]. Also, Cohen’s d was evaluated for the t-test as following: d = 0.2 small; d = 0.5 medium; d = 0.8 large; and d = 1.3 very large [24]. The level of statistical significance was set at *p* < 0.05.

## 3. Results

**Figure 5:**
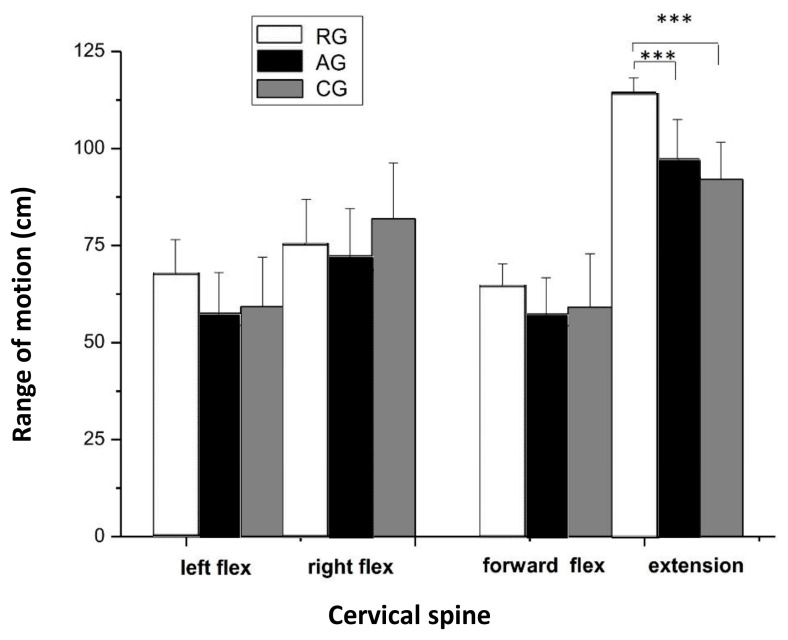
Cervical joint range of motion (means ± SD) during left and right lateral flexion, forward flexion, and extension. RG = rhythmic gymnasts, AG = artistic gymnasts, and CG = control group; *** *p* < 0.001.

The RG athletes had a significantly greater ROM in head extension compared to the AG athletes and CG participants (*p* < 0.001, η_p_^2^ = 0.454). However, no differences were found among the three groups in left (η_p_^2^ = 0.050) and right (η_p_^2^ = 0.065) lateral flexion, forward flexion (η_p_^2^ = 0.011), and rotation (η_p_^2^ = 0.072) (Figure 5).

**Figure 6:**
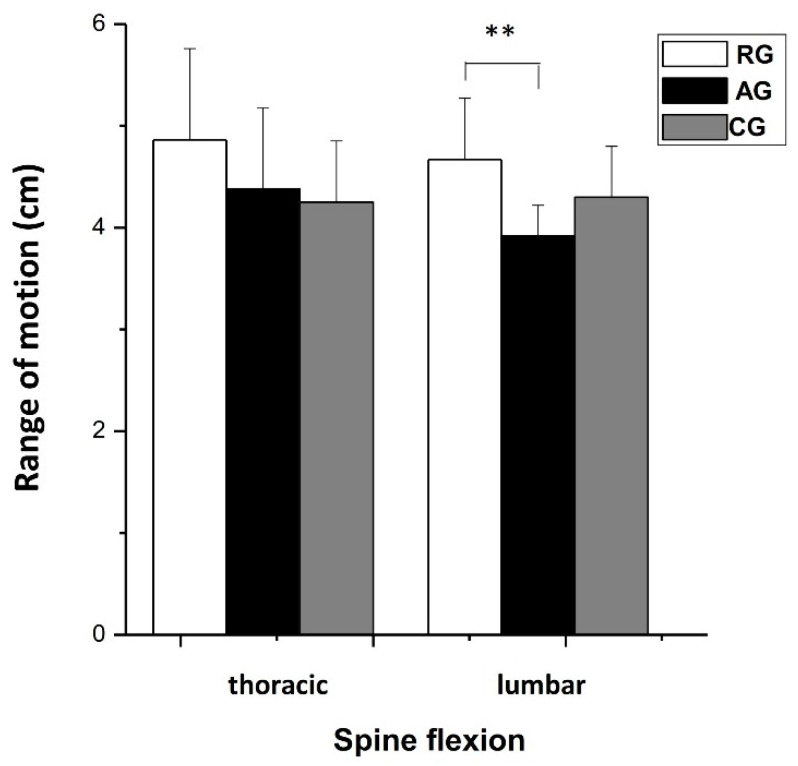
Thoracic and lumbar spine range of motion (means ± SD). RG = rhythmic gymnasts, AG = artistic gymnasts, and CG = control group; ** *p* < 0.01.

The ROM at the lumbar spine was significantly greater (*p* < 0.01, η_p_^2^ = 0.437) in the RG athletes compared with AG athletes. No significant differences were found among the three groups in the ROM of the thoracic spine (η_p_^2^ = 0.064); the flexion of the trunk from the standing position, from C7 to S1 (η_p_^2^ = 0.055); and the lateral bending of the trunk (left–right lateral flexion, η_p_^2^ = 0.058) (Figure 6).

**Figure 7:**
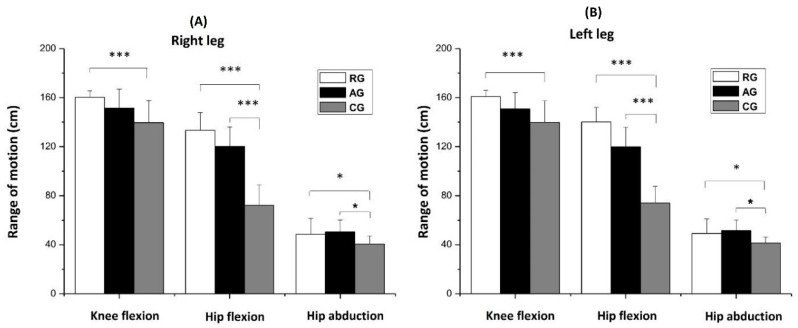
Range of motion (means ± SD) of the knee flexion, hip flexion, and hip abduction of the right leg (**A**) and left leg (**B**). RG = rhythmic gymnasts, AG = artistic gymnasts, and CG = control group; * *p* < 0.05 and *** *p* < 0.001.

The RG group showed significantly greater ROM in knee flexion (*p* < 0.001; right leg: η_p_^2^ = 0.344, left leg: η_p_^2^ = 0.320), hip flexion (*p* < 0.001; right leg: η_p_^2^ = 0.913, left leg: η_p_^2^ = 0.902), and hip abduction (*p* < 0.05, right leg: η_p_^2^ = 0.194, left leg: η_p_^2^ = 0.196) compared to the CG. Also, the ROM was greater in hip flexion (*p* < 0.001) and hip abduction (*p* < 0.05) in the AG group in comparison to the CG. However, no significant differences were found between the RG and AG groups. These results were similar for both the right and left leg (Figure 7).

**Figure 8:**
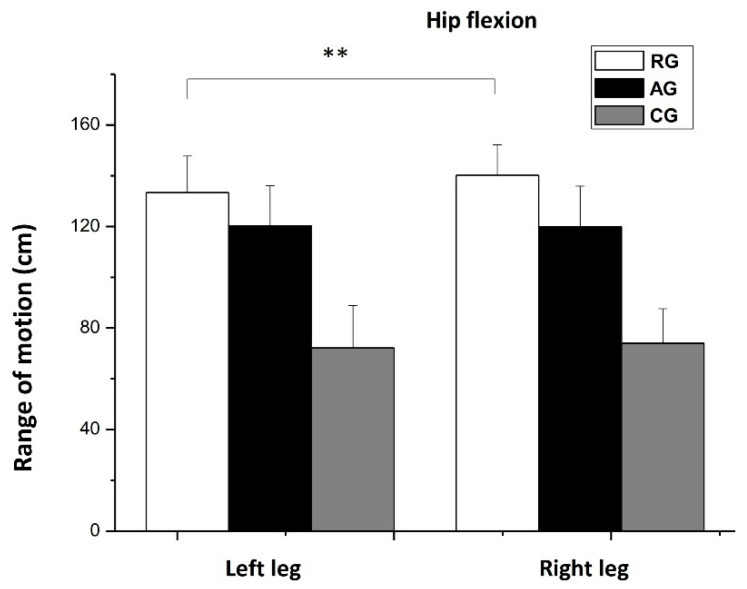
Range of motion (means ± SD) of the hip flexion (hamstring muscles). Comparison between right and left legs among the three groups. RG = rhythmic gymnasts, AG = artistic gymnasts, and CG = control group; ** *p* < 0.01.

Surprisingly, a significant difference was observed in the ROM of hip flexion in the RG group, where the right leg was significantly higher, compared to the left leg (*p* < 0.01, d = 0.5). No significant differences were observed between the left and right leg in hip flexion for the AGs (d = 0.1) and CG (d = 0.1) (Figure 8).

The results of the absolute and relative isokinetic muscle strength between the RGs, AGs, and CG are shown in Table 2. In the absolute isokinetic concentric muscle strength of the quadriceps, the CG had significantly greater strength at 180°·s^−1^ (η_p_^2^ = 0.221) and 300°·s^−1^ (η_p_^2^ = 0.238), compared to the RGs (*p* < 0.01 and *p* < 0.05) and AGs (*p* < 0.05, respectively) groups. No significant differences were found among the three groups, at the slow angular velocity (60°·s^−1^, η_p_^2^ = 0.058). However, the relative muscle strength of the quadriceps in the RG and AG groups was significantly greater at 60°·s^−1^ compared to the CG (*p* <0.001 and *p* <0.01, respectively; η_p_^2^ = 0.221) and between the AGs and CG at 180°·s^−1^ (*p* < 0.05, η_p_^2^ = 0.153). Nevertheless, no significant differences were shown between the RG and CG groups. In knee flexion there were no significant differences in absolute muscle strength between the groups (60°·s^−1^: η_p_^2^ = 0.019, 180°·s^−1^: η_p_^2^ = 0.032, 300°·s^−1^: η_p_^2^ = 0.093) as well as in the H:Q ratio (60°·s^−1^: η_p_^2^ = 0.026, 180°·s^−1^: η_p_^2^ = 0.169, 300°·s^−1^: η_p_^2^ = 0.028). However, a significant difference was observed in the relative muscle strength at 60°·s^−1^ between RGs and the CG (*p* < 0.05, η_p_^2^ = 0.184).

## 4. Discussion

The purpose of this study was to examine and compare the joint mobility and muscle strength of the spine and lower extremities in prepubertal elite athletes in rhythmic gymnastics, artistic gymnastics, and untrained subjects of the same age. The results of the study showed that the PROM in cervical extension was notably higher in the RG compared to the AGs and CG. Moreover, both athlete groups, RG and AG, exhibited significantly greater PROM in knee flexion, hip flexion, and hip abduction in comparison to the CG. Additionally, the relative muscle strength of the quadriceps in the RGs and AGs was significantly higher than in the CG.

In agreement with previous studies, the RGs are thinner than average [39] and they have lower BMI than untrained peers [40,41], while the AGs have a mean height score below the 50th percentile [42]. In addition, young AGs appear to have some lower anthropometric characteristics compared to other athletes (e.g., swimmers) and non-athletes [43]. Anthropometric characteristics are significant predictors to performance in rhythmic gymnastics [44] and artistic gymnastics [45].

Generally, the torque–velocity relationship in young populations indicates a similar, adult-like pattern: as the angular velocity increased the peak torque decreased. Quadriceps muscle strength is greater than hamstrings at all angular velocities and in all groups. Several authors have mentioned that in these ages there are no differences between boys and girls. Basa et al. [46] suggested that long-term gymnastic training in prepubertal boys was associated with increased torque of the knee extensors but not of the knee flexors, which is consistent with the findings of our study. Thus, training in gymnastics, artistic and rhythmic, in prepubescent ages may not be associated with increased torque of the knee flexors. Several points of this finding should be taken under consideration. First, muscle strength can be influenced by body size [47] and participants in the CG were taller and heavier than athletes. It is known that individuals with higher body mass have greater absolute strength. This study showed that RGs had greater relative muscle strength because of their lower body mass. There is the possibility that the participants of the three groups under observation were in different levels of sexual maturity (Tanner stage); for example, athletes could be in stage I, whereas the participants of the CG could be in stage II. Moreover, it is well known that elite RGs have an observed delay in pubertal development [48] and skeletal maturation [49]. Second, at this age, physical activities are part of the child’s everyday-life activity and so participants in the CG cannot be considered fully sedentary, as is mentioned in another study [50]. All children have taken part in physical education classes at school, which can increase their strength [51]. Finally, in rhythmic gymnastics the high scores in strength tests do not seem to be related to success, as elite RGs do not produce especially high scores in the above tests [52].

RGs were more flexible than both the AGs and the CG. In rhythmic gymnastics, lean body mass and composite measures of joint mobility are significant correlates of attainment [41] and flexibility correlates significantly to performance [53,54]. Some studies have shown that AGs are characterized by a special physical characteristic in comparison to their non-athletic peers or athletes of other sports [55,56]. The selection of the athletes performed by the coaches relates to the demands of the sports (i.e., muscle strength and power and muscle endurance), in order to present mechanical advantages in performing the exercises. Most body exercises in rhythmic gymnastics are based on ballet and are performed by an “en de hors” (turn-out) turn of joints [57]; so, from their early training years they practice performing joints turn-outs, unlike the artistic gymnastics. Probably, in artistic gymnastics a turn-out joint mobility does not help the execution of specific elements. Based on the code, artistic gymnastic elements use less “en de hors” turn of joints in hip abduction. This could be the reason why, in assessing right hip abduction, AGs have higher joint mobility. Spinal mobility was significantly higher for RGs. According to the code in every body group (jumps, balances, turns, and flexibility), elements with back bends obtain a higher evaluation [58]. Back extensions and split leaps with back extensions are common elements in this type of gymnastics, so this emphasis on flexibility and repetitive demand of back extensions places the lumbar spine of the athletes at risk [15]. Increased lumbar lordosis can result in anterior pelvic tilt, which due to repeated loads may lead to spondylolysis [59]. The close relationship between spondylolysis and increased pelvic tilt (i.e., increased lumbosacral angle) has been observed in competitive gymnasts [60]. Therefore, it would be preferable to pay attention to the whole spine or better to its coordination, rather than to the hypermobility of one segment.

Exercises and training programs guided by the rules of evaluation increase mobility in the bending angle of the part of the spine which is already flexible enough and not in the part which shows limited mobility. This refers to the limited mobility of the thoracic spine and the excessive mobility of the lumbar spine. Repetitive hyperextensions in the lumbar spine contribute to overloading the spine. However, it must be pointed out that the spine consists of layers of muscles, some of which have the ability to shorten and others to lengthen [61]. Hence, in terms of muscle strength and mobility, it is hard to find the right way to treat it.

### Limitations and Future Directions

This study had several limitations. The biological age was not examined; therefore, there is a possibility that the participants of the three groups under observation were in different levels of sexual maturity (Tanner stage), where the results of the study may have been affected. Also, the participants were prepubertal girls; therefore, the extrapolation of our finding into a general gymnastic should be performed with caution, and care should be taken when applying the study results. Another limitation is that the muscle strength was measured using an isokinetic dynamometer, in the muscle groups of quadriceps and hamstring, and in concentric knee flexion/extension. Therefore, further research should focus on other muscle groups, as well as eccentric strength measurements. Also, future research should include functional strength tests that are related to the sport and important for success in specific gymnastic performance.

## 5. Conclusions

The present study examined the muscle strength and joint range of motion of the back and lower extremities in prepubertal female RGs, AGs, and a CG. The results of the present study have shown that RGs were more flexible than AGs, which could be a result of their specific sport training and the requirements of the sport. Significant differences were found between left and right leg hip flexion in RGs. This study has also shown that gymnastics training in prepubertal ages, both rhythmic and artistic, can improve neuromuscular function, i.e., technique, and increase relative muscle strength. Therefore, it is essential to note that when evaluating children within the developmental ages, especially those involved in sports, the type of muscle strength to be assessed should be specified.

## Figures and Tables

**Table 1:** Physical characteristics of the participants (means ± SD).

	Rhythmic Gymnasts (RG) (n = 18)	Artistic Gymnasts (AG) (n = 18)	Control Group (CG) (n = 18)
Age (years)	11.14 ± 0.70	11.27 ± 0.99 +	10.55 ± 0.42
Height (cm)	142.6 ± 5.81	139.6 ± 5.85	145.33 ± 6.95 ++
Body mass (kg)	31.2 ± 3.63	31.7 ± 3.21	42.1 ± 8.21 ### +++
Body mass index (Kg/m^2^)	15.22 ± 1.76	16.67 ± 1.85	20.06 ± 2.90 ### +++
Years in training (years)	4.03 ± 0.8	4.40 ± 0.5	0
Hours of daily training (hours)	3.83 ± 0.65 *	3.42 ± 0.28	0

* *p* < 0.05 comparison between RGs and AGs, + *p* < 0.05 comparison between the CG and AGs, ++ *p* < 0.01 comparison between the CG and AGs, +++ *p* < 0.001 comparison between the CG and AGs, and ### *p* < 0.001 comparison between the CG and RGs.

**Table 2:** Quadriceps and Hamstring peak torque values (Nm) and relative body mass values (Nm·kg^−1^BW) at angular velocities of 60, 180, and 300°·s^−1^. Comparison between RGs, AGs, and CG (mean ± SD).

		RG	AG	CG
Quadriceps absolute values (Nm)	60°·s^−1^	82.33 ± 13.3	79.85 ± 11.7	89.89 ± 25.0
	180°·s^−1^	48.28 ± 8.9 ++	53.92 ± 7.9 #	63.11 ± 16.6
	300°·s^−1^	34.28 ± 6.1 +	34.85 ± 4.7 #	44.17 ± 12.2
Hamstring absolute values (Nm)	60°·s^−1^	41.22 ± 7.0	41.31 ± 9.1	44.00 ± 11.7
	180°·s^−1^	26.78 ± 5.1	27.31 ± 7.7	29.56 ± 7.0
	300°·s^−1^	18.00 ± 3.7	17.54 ± 5.9	21.56 ± 7.1
Quadriceps relative to body mass values (Nm·kg^−1^BW)	60°·s^−1^	2.63 ± 0.25 +++	2.51 ± 0.26 ##	2.14 ± 0.40
	180°·s^−1^	1.55 ± 0.19	1.69 ± 0.14 #	1.50 ± 0.24
	300°·s^−1^	1.09 ± 0.14	1.09 ± 0.66	105 ± 0.19
Hamstring relative to body mass values (Nm·kg^−1^BW)	60°·s^−1^	1.32 ± 0.19 +	1.29 ± 0.22	1.07 ± 0.30
	180°·s^−1^	0.86 ± 0.19	0.85 ± 0.20	0.71 ± 0.18
	300°·s^−1^	0.58 ± 0.20	0.54 ± 0.16	0.51 ± 0.14
Hamstings:Quadriceps ratio	60°·s^−1^	50.07 ± 5.3	51.73 ± 8.3	48.95 ± 7.5
	180°·s^−1^	55.34 ± 5.7	50.65 ± 9.7	46.84 ± 7.8
	300°·s^−1^	52.51 ± 6.1	50.33 ± 12.5	48.81 ± 7.3

# *p* < 0.05 comparison between AGs and CG, ## *p* < 0.01 comparison between AGs and CG, + *p* < 0.05 comparison between RGs and CG, ++ *p* < 0.01 comparison between RGs and CG, and +++ *p* < 0.001 comparison between RGs and CG.

## Data Availability

The data presented in this study are available on request from the corresponding author. The data are not publicly available due to privacy restrictions.
